# Interaction of Alcohol Consumption and *ABCG2* rs2231142 Variant Contributes to Hyperuricemia in a Taiwanese Population

**DOI:** 10.3390/jpm11111158

**Published:** 2021-11-07

**Authors:** I-Chieh Chen, Yen-Ju Chen, Yi-Ming Chen, Hsueh-Ju Lin, Ying-Cheng Lin, Jui-Chun Chagn, Pei-Chun Chen, Ching-Heng Lin

**Affiliations:** 1 Department of Medical Research, Taichung Veterans General Hospital, Taichung 40705, Taiwan; icchen@vghtc.gov.tw (I.-C.C.); aoaichen@gmail.com (Y.-J.C.); ymchen1@vghtc.gov.tw (Y.-M.C.); cheryllin520@gmail.com (H.-J.L.); 2 Division of Allergy, Immunology and Rheumatology, Department of Internal Medicine, Taichung Veterans General Hospital, Taichung 40705, Taiwan; 3 Institute of Clinical Medicine, National Yang Ming Chiao Tung University, Taipei City 11221, Taiwan; 4 Institute of Biomedical Science and Rong Hsing Research Center for Translational Medicine, National Chung Hsing University, Taichung 402202, Taiwan; 5 School of Medicine, National Yang Ming Chiao Tung University, Taipei City 11221, Taiwan; 6 Division of Gastroenterology and Hepatology, Department of Internal Medicine, Taichung Veterans General Hospital, Taichung 40705, Taiwan; ethankevin516@gmail.com; 7 Department of Obstetrics and Gynecology and Women’s Health, Taichung Veterans General Hospital, Taichung 40705, Taiwan; r.juichun@gmail.com; 8 Department of Mathematics and Information Education, National Taipei University of Education, Taipei City 10671, Taiwan; 9 Department of Public Health, College of Medicine, Fu Jen Catholic University, New Taipei City 24205, Taiwan; 10 Department of Health Care Management, National Taipei University of Nursing and Health Sciences, Taipei City 112303, Taiwan; 11 Department of Industrial Engineering and Enterprise Information, Tunghai University, Taichung 40704, Taiwan; 12 Institute of Public Health and Community Medicine Research Center, National Yang Ming Chiao Tung University, Taipei City 11221, Taiwan; 13 Department of Internal Medicine, China Medical University Hospital, Taichung 404332, Taiwan

**Keywords:** hyperuricemia, serum uric acid levels, alcohol consumption, *ABCG2* rs2231142

## Abstract

Background: *ABCG2* rs2231142 is an important genetic factor that contributes to the development of gout and hyperuricemia (HUA). Epidemiologic studies have demonstrated that lifestyle risk factors of HUA (e.g., alcohol consumption) and genetic predisposition (e.g., *ABCG2* gene) together, contribute to enhanced serum uric acid levels. However, the interaction between *ABCG2* rs2231142, alcohol consumption, and HUA in the Taiwanese population is still unclear. Therefore, this study investigated whether the risk of HUA is associated with *ABCG2* rs2231142 variants and how this is affected by alcohol consumption. Method: study subjects were selected from the participants of the Taiwan Biobank database. Overall, 114,540 participants aged 30 to 70 years were enrolled in this study. The interaction between *ABCG2* rs2231142, alcohol consumption, and serum uric acid (sUA) levels was analyzed by multiple logistic regression models. Results: the prevalence of HUA was 32.7% and 4.4 % in the male and female populations, respectively. In the whole study population, the minor T allele of *ABCG2* rs2231142 was significantly associated with HUA risk, and the occurrence of HUA was high in TT genotype and TG genotype. The risk of HUA was significantly increased by the combined association of *ABCG2* rs2231142 and alcohol consumption for TG/TT genotype compared to the GG genotype (wild-type genotype), especially among women. Conclusion: the *ABCG2* rs2231142 is a crucial genetic locus for sUA levels in the Taiwanese population and our findings revealed that alcohol consumption combined with the *ABCG2* rs2231142 risk allele contributes to increased HUA risk.

## 1. Introduction

Uric acid is a product of purine metabolism in humans. A high blood concentration of uric acid is known as hyperuricemia (HUA, uric acid levels > 7.0 mg/dL), which can be caused by an imbalance in uric acid uptake, synthesis, or excretion [1,2], and is related to gout, cardiovascular disease, chronic kidney disease, type 2 diabetes mellitus [2,3,4,5], and metabolic related diseases [6,7]. HUA has received increasing attention as a major public health problem in Taiwan due to its high prevalence and the associated increases in the risks of various diseases. The cause of HUA is multi-factorial, including age, gender, obesity, diet, alcohol consumption, insulin resistance, hypertension, and medication [2,8].

Several genome-wide association studies (GWAS) of serum uric acid (sUA) have identified that sUA is under strong genetic control and more than ten sUA associated genes have been identified [9,10], such as ATP-binding cassette subfamily G member 2 (*ABCG2*), glucose transporter type 9(*GLUT9*, also known as *SLC2A9*), and urate anion transporter1 (*URAT1*, also known as *SLC22A12*). In a genome-wide study, *ABCG2* rs2231142 displayed strong evidence of an association with sUA levels (*p* < 10^−60^) [9]. Furthermore, recent GWAS have also reported a strong association between *ABCG2* rs2231142 and sUA concentrations in a Chinese population (*p* = 3.341 × 10^−42^) [11]. In the Taiwanese population, the *ABCG2* rs2231142 gene had a significant association with HUA (OR = 2.15, *p* < 0.001) after adjustment for potential confounders [12].

*ABCG2* encodes a high-capacity urate efflux transporter, located in a gout-susceptibility locus (MIM 138900) on chromosome 4q [13], and some genetic variants can increase uric acid levels [14,15]. The *ABCG2* rs2231142 single nucleotide polymorphism (SNP) in exon 5, and this missense mutation leads to a Glu141Lys amino acid substitution (Q141K) [15,16], which accounted for 0.57% of the variation in serum urate [17]. To the best of our knowledge, *ABCG2* rs2231142 SNPis one of the most significant genetic variants associated with HUA in the Asian population [10,18]. Recent studies have indicated the Q141K polymorphism was associated with a reduced *ABCG2* protein surface expression [19,20]. Kaszaet al. found that nonsense mutations on one allele result in a 50% reduction in *ABCG2* protein expression in the human erythrocytes [19]. Meanwhile, the *ABCG2* variant (Q141K) also reduced *ABCG2* adenosine triphosphatase (ATPase) activity and modified the transporter activity of *ABCG2* [20,21]. The functional study of rs2231142 has shown that it causes a 53% reduction in the rate of *ABCG2*-mediated urate transport compared with wild-type [15,22]. Therefore, people carrying the *ABCG2* rs2231142 T allele are more likely to have HUA.

Previous studies revealed that the T allele of *ABCG2* rs2231142 was associated with increased sUA levels and its frequency is approximately three-fold higher in East Asian populations compared to European populations [10,18]. SNP rs2231142 is significantly associated with HUA and gout risk in East Asia compared to other sUA-risk genes [10,18]. In the Taiwanese population, the prevalence of HUA in males and females is 43.71% and 27.4% [23], respectively, which is higher than in other ethnic groups [2]. Recently, epidemiologic studies have demonstrated that lifestyle risk factors of HUA (e.g., alcohol consumption) and genetic predisposition (e.g., *ABCG2* gene) together, contribute to enhanced sUA levels [22,24,25]. These studies have shown a significant association between alcohol drinking and HUA, which may partially explain the incidence and prevalence of gout [24,26]. A recent study showed that the prevalence of HUA in males was 11.9% in non-drinkers, 12.6% in moderate drinkers, and 16.3% in heavy drinkers (*p* < 0.001) in a general population from rural China [27]. In females, the rates were 6.3% in non-drinkers, 8.1% in moderate drinkers, and 6.6% in heavy drinkers (*p* = 0.818). In a Taiwanese cohort study, *ABCG2* rs2231142 was associated with tophaceous gout across the alcohol consumption, with a stronger association in everdrinkers (OR = 25.05) than in current drinkers (OR = 12.69) [28]. To date, few studies have investigated the association between alcohol consumption and *ABCG2* rs2231142 variants in a general population in Taiwan even though it is clearly associated with UA. Therefore, in this study, we examined whether the different joint effects of alcohol consumption and *ABCG2* rs2231142 risk allele in males and females contribute to increased HUA risk.

## 2. Materials and Methods

### 2.1. Data Source and Study Sample

This study was conducted using the Taiwan Biobank (TWB), which gathered information and specimens from a convenience sample of Taiwanese volunteer participants in recruitment centers across Taiwan. All of the participants provided informed consent. Detailed information on the program and data access is available from the website of the TWB [29,30]. Our study cohort was composed of 114,540 individuals aged 30 to 70 years. Genotyping information, serum uric acid reports, demographic information, medical history, lifestyle modality, body fat evaluation (including BMI, waist circumference, and body fat percentage), and biochemical reports (including serum creatinine, cholesterol, and fasting glucose level), were all identified from the database. This research project was approved by the ethics committee of Taichung Veterans General Hospital Institutional Review Board (IRB no. CE16270B-1). The study was conducted in accordance with the principles of the Declaration of Helsinki and the Good Clinical Practice Guidelines, and all the participants provided informed consent. All available data were acquired from TWB, which collects specimens and information in a complete and standardized procedure to fit researchers’ needs in different fields [30,31,32].

### 2.2. SNP Genotyping and Quality Controls

The blood DNA samples from TWB participants of adults aged 30 to 70 years were genotyped using the custom Taiwan Biobank 2.0 SNP chip and an Axiom Genome-Wide Array Plate System (Affymetrix, Santa Clara, CA, USA) at the National Center for Genome Medicine in Academia Sinica, Taiwan [30,32]. TWB used the Affymetrix Power Tools (APT) and performed a standard quality control procedure to exclude SNPs with low call rates (<99%), with a *p* value for the Hardy-Weinberg equilibrium test of <1.0 × 10^−4^ for the controls and a minor allele frequency of <0.01. The Affymetrix TWB 2.0 SNP chip contained 653,291 SNPs and was designed specifically for Taiwan’s Han Chinese population. Details on the TWB can be found on its official website (TaiwanView: http://taiwanview.twbiobank.org.tw) [33]. SNPs on the X and Y chromosomes, as well as those on mitochondrial DNA, were also included for data release [30]. PLINK was used for analysis and a quality control procedure was performed to exclude markers that failed Hardy–Weinberg equilibrium tests with a *p* value < 1 × 10^−6^, minor allele frequency <0.01, and a genotyping call rate less than 90% [34].

### 2.3. Data Collection and Outcome

The primary outcome was serum uric acid level, measured by means of a uricase method, at the baseline of each study [30]. The sUA level was measured using an Architect i2000SR Analyzer (Abbott Diagnostics, Abbott Park, Chicago, IL, USA). There are 114,540 individuals with genotyping information in the data source, and 3 were excluded without serum uric acid values. Finally, we recruited 13,492 and 3254 participants with HUA (sUA levels  > 7.0 mg/dL; without gout disease history) in the male and female populations, respectively, from the TWB database. A total of 27,773 males and 70,018 females were used as normal controls (sUA levels  ≤ 7.0 mg/dL with neither gout nor HUA).Relevant laboratory examinations and lifestyle data of TWB subjects were provided by the TWB database. Covariates were evaluated as follows: participants’ demographic (including gender, age, and marital status), personal health behaviors (including habits of alcohol drinking, smoking, and routine physical activity), physical examination (including body mass index, and waist circumference [WC, cm]), and blood and urine tests (including blood pressure [BP, mmHg], total cholesterol [TC, mg/dL], triglyceride [TG, mg/dL], high-density lipoprotein [HDL, mg/dL] cholesterol, low-density lipoprotein [LDL, mg/dL] cholesterol, fasting glucose [FG, mg/dL], and creatinine [mg/dL]).Body mass index (BMI) was calculated as weight (kilograms, kg) divided by height (meters, m^2^) squared, and overweight was defined as BMI ≥ 24 kg/m^2^. Central obesity was defined as WC ≥ 90 cm in men or ≥ 80 cm in women. Hypertension was defined as BP ≥ 140/90 mmHg. Regarding blood and urine test results, suspected hyperlipidemia was defined as TC ≥ 200 mg/dL, TG ≥ 150 mg/dL, HDL < 40 mg/dL in men or <50 mg/dL in women, and LDL > 100 mg/dL. Suspected diabetes was defined as FG ≥ 100 mg/dL. Lifestyle factors considered in this study included cigarette smoking, alcohol consumption, and physical activity, which were obtained by a questionnaire. The smoking status was dichotomized as a current- or ever-smoker versus a non-smoker. The alcohol-consumption status was dichotomized as a current- or ever-drinker versus a non-drinker. For physical activity, subjects were dichotomized as non-sedentary versus sedentary [35].

### 2.4. Statistical Analysis

All the statistical analyses were performed using the SAS version 9.4 software (SAS Institute Inc., Cary, NC). The characteristics of the continuous variables were expressed as means ± standard deviations and were analyzed using Student’s t-tests or analysis of variance (ANOVA). Comparisons of categorical variables were analyzed using the Chi-square test. The interaction between *ABCG2* rs2231142 and alcohol consumption, and hyperuricemia was analyzed by multiple logistic regression models. Meanwhile, the association between *ABCG2* rs2231142 and alcohol consumption in HUA was analyzed using a logistic regression model to adjust for potential confounders. Odds ratios (OR) and 95% confidence interval (95% CI) were calculated. A *p* value less than 0.05 was considered to be statistically significant.

## 3. Results

### 3.1. Baseline Characteristics of Study Population

A total of 114,537 participants, including 41,265 males and 73,272 females, were enrolled in this study. The prevalence of HUA was 32.7% and 4.4% in the male and female populations, respectively. The basic characteristics of participants are shown in Table 1. The ratio of HUA was significantly higher in postmenopausal women aged 55 to 70 years (72.6%, *p* < 0.001) in the HUA cases. In contrast, the ratio of HUA was higher in males aged 30 to 45 years (27.0%, *p* < 0.001), compared to all male participants with HUA. The individuals in the HUA group had higher level of BMI, WC, BP, TC, TG, and LDL levels, but lower levels of HDL, compared to controls (*p* < 0.001). Moreover, there was no significant difference in tobacco smoking, chewing betel nut or body fat percentage between the case and control groups in females.

### 3.2. Distribution and Association of ABCG2 rs2231142 Variants in HUA

The distribution of *ABCG2* rs2231142 variants are shown in Table 2. In the male population, the genotype frequencies of *ABCG2* rs2231142 among HUA cases were 14.3% (TT), 48.5% (TG), and 37.2% (GG), while the genotype frequencies were 7.9% (TT), 40.3% (TG), and 51.7% (GG) in the controls. In female, the genotype frequencies of *ABCG2* rs2231142 among HUA case were 16.2% (TT), 50.0% (TG), and 33.9% (GG).The minor T allele frequency of *ABCG2* rs2231142 was present in 38.5% of the HUA population compared with 28.1% of the control group in males and 41.1% of the HUA population in females compared to 31.1% in the control group.The distribution of *ABCG2* variants showed statistical significance in different genotypes (*p* < 0.001) between the HUA cases and controls in both males and females. Additionally, the risk of HUA was significantly associated with *ABCG2* rs2231142 risk T allele, and both TT and TG genotypes contributed to an increased risk of HUA in men (TT: OR = 2.49, 95% CI: 2.33–2.67, *p* < 0.001; TG: OR = 1.67, 95% CI: 1.59–1.74, *p* < 0.001) and women (TT: OR = 2.34, 95% CI: 2.10–2.61, *p* < 0.001; TG: OR = 1.64, 95% CI: 1.51–1.77, *p* < 0.001).

### 3.3. Association between ABCG2 rs2231142 and Alcohol Consumption in HUA

**Figure 1:**
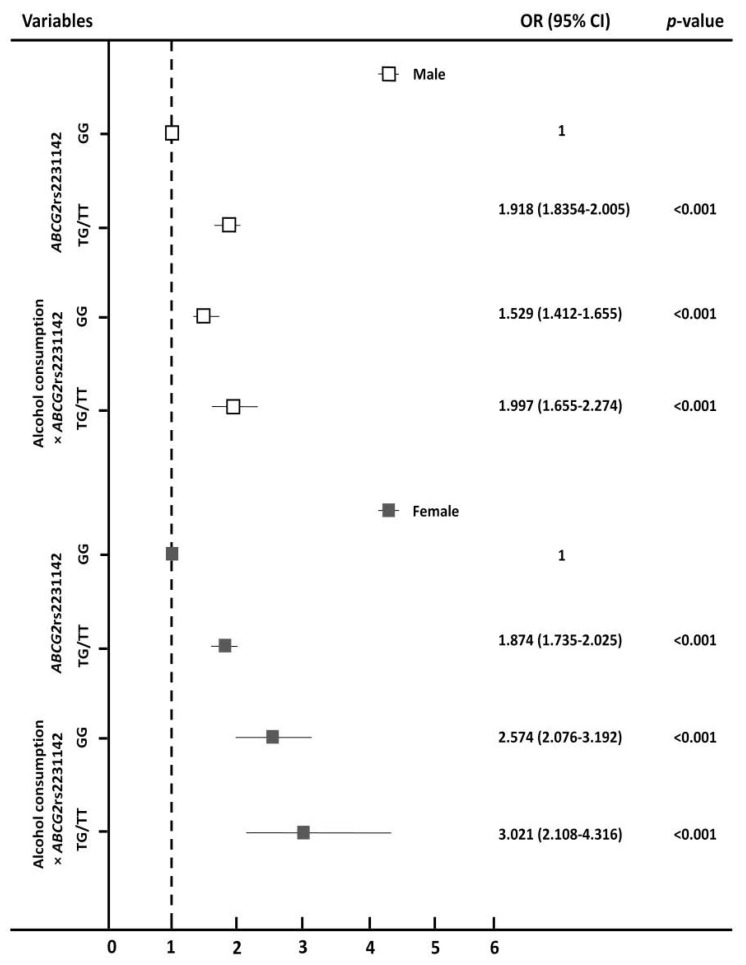
Odds ratios for the association between *ABCG2* rs2231142 and alcohol consumption with hyperuricemia. Dotted line represents an OR of 1. Error bars represent the 95% confidence interval of the odds ratios. The *p*-values were generated using logistic regression.

We assessed the statistical significance of the interaction between alcohol consumption and *ABCG2* rs2231142 variants by using the multiple logistic regression model. As shown in Table 3, the risk of HUA markedly increased due to the interaction of *ABCG2* rs2231142 and alcohol consumption. Compared to the subjects with GG, the risk for HUA increased by 118% in men (OR = 2.183, 95% CI: 2.059–2.315, *p* < 0.001) and by 209% in women (OR = 3.096, 95% CI: 2.403–3.988, *p* < 0.001) with the TG/TT genotype. We further characterized the association between alcohol consumption and *ABCG2* rs2231142 variants along with their interactions (Figure 1). The results indicated that the risk of HUA was significantly associated with *ABCG2* rs2231142 risk T allele, as the risk for HUA increased by 91.8% in men (OR = 1.918, 95% CI: 1.834–2.005, *p* < 0.001) and 87.4% in women (OR = 1.874, 95% CI: 1.735–2.025, *p* < 0.001) with TG/TT genotype. Of note, the risk of HUA was significantly increased by the interaction between *ABCG2* rs2231142 TG/TT genotypes and alcohol consumption in females (OR = 3.021, 95% CI: 2.108–4.316, *p* < 0.001).

## 4. Discussion

In this cohort study, we confirmed the importance of the *ABCG2* rs2231142 T allele with a high risk of HUA. We showed that alcohol consumption combined with *ABCG2* rs2231142 risk allele contributes to increased HUA risk in both genders, especially women. In our findings, the minor T allele of *ABCG2* rs2231142 was a critical risk factor for HUA and the risk of HUA was significantly increased by the interaction of *ABCG2* rs2231142 and alcohol consumption for TG/TT genotype (OR = 1.997, 95% CI: 1.655–2.275, *p* < 0.001 in males, OR = 3.021, 95% CI: 2.108–4.316, *p* < 0.001 in females). We demonstrated that *ABCG2* rs2231142 contributed to the risk of HUA, especially for the TG/TT genotype. This is consistent with other Asia population studies [16,36], suggesting that patients carrying the T allele had a higher frequency of HUA than those with the *ABCG2* rs2231142 GG allele. The combined genetic effects could explain some proportion of inter-individual variation in sUA.

Genetic factors play an important role in HUA risk, but these factors do not change within an individual. We found significant interaction between *ABCG2* rs2231142 and alcohol consumption in Taiwanese people, though we did not have access to details about the type of alcohol consumed. Alcohol has been recognized as a potential risk factor for HUA and is considered a trigger for gouty arthritis and recurrent gout attacks [24,37]. The Third US National Health and Nutritional Examination Survey (NHANES III, 1988–1994) study showed that sUA increased with alcohol consumption and decreased with increasing dairy intake. Moreover, alcohol consumption(particularly beer and liquor consumption)increased sUA levels [38]. Similarly, Nakayama et al. indicated that alcohol consumption (3.5 × 10^−4^ mg/dL) was a significant factor in increasing sUA, and a regression analysis revealed that “552.1 g/week alcohol intake as pure ethanol” was equivalent to a 25% decrease in *ABCG2* function in terms of ability to increase sUA levels [39]. Moreover, one meta-analysis by Tu et al. showed that alcohol consumption and *ABCG2* rs2231142, independently and jointly, were associated with the risk of chronic tophaceous gout [28].The effect of alcohol is, in part, related to increased urate production by activation of adenine nucleotide turnover, which is due to enhanced turnover of ATP during the conversion of acetate to acetyl-CoA as part of the metabolism of ethanol [40]. Furthermore, acute alcohol consumption causes lactate production. Lactate is an antiuricosuric agent that reduces renal urate excretion and exacerbates HUA [26]. In addition, part of the association of alcohol intake with HUA is likely related to the high lead content in certain liquors, especially in port wines. Lead causes a marked increase in sUA by impairing urate excretion [26].

The prevalence of HUA ranges from 13.3% to 21.6% and varies by sex and among countries or regions [2,41]. Local differences are apparent within countries, likely influenced by environmental, climatic, economic, and dietary factors. In this study, the prevalence of HUA was 32.7% and 4.4 % in the male and female populations, respectively. The prevalence of HUA increased with age in women but was stably high in men. We uncovered an extra layer of interaction between sex, age, and alcohol consumption on sUA levels. Our data showed that *ABCG2* rs2231142 was significantly associated with UA levels in senior females, which may be explained by menopause and other age-related factors influencing female hormones [42,43]. According to Choi’s findings, postmenopausal hormone use is associated with lower uric acid levels among postmenopausal women. In postmenopausal women, the increased levels of sUA are thought to be caused by a change in renal urate elimination associated with the loss of estrogens [44,45]. Estrogens promote renal uric acid excretion and decrease the level of sUA by suppressing the protein levels of URAT1 and GLUT9 in the proximal tubule, and that of urate efflux transporter *ABCG2* [46]. Furthermore, administration of estrogen therapy to postmenopausal women was shown to decrease serum uric acid levels [44,45]. Our data are consistent with previous cross-sectional studies that found age-related increases in sUA among women but not such variation among men [47,48,49]. A study based on 3013 female residents of Tecumseh, MI [48] and a study based on 254 women in the UK [49] reported a rise in serum urate levels after age 50 to 54 years with a subsequent plateau. In addition, a study based on 18,324 Japanese females reported increasing uric acid levels up to the age of 70 years by Akizuki et al. In this study, we found that serum uric acid levels among women increased from age 45 to 55 onwards and the increase extended up to the highest age category of 70 years of age. The age-associated increase in sUA may be explained by other age-related factors such as renal function, diuretic use and hypertension. Whether these factors also affect the risk of HUA more so in women than among men remains to be examined in prospective studies.

The mechanism through which the *ABCG2* gene influences serum urate levels is not fully understood. However, it has been reported that it codes for the ATP-binding cassette super-family G member 2 regulatory sUA via physiologically important roles in both renal and intestinal urate excretion [14,15,50,51,52,53]. The function of *ABCG2* protein as an important urate transporter was inferred from genome-wide study and subsequent functional research. *ABCG2* rs2231142 demonstrated a strong association with HUA, and highlighted the role of rs2231142 in the pathogenesis of reduced cellular urate efflux, HUA, and early-onset gout. *ABCG2* plays a physiological role of urate homeostasis in the human body through both renal and extrarenal urate excretion via the bile or intestine [14]. However, it is difficult to obtain accurate non-invasive measurements of intestinal/biliary urate secretion in humans because the secreted urate is largely metabolized by the bacterial flora in the intestine. Hence, Hosomi and colleagues demonstrated that the role of *ABCG2* in extrarenal urate secretion was revealed in animal models using the in situ intestinal “closed-loop” perfusion method [52,53]. They demonstrated that, besides the substantial fraction of renal urate elimination, there is direct urate excretion via the intestine, and only minor urate excretion via the bile [52,53].

*ABCG2* rs2231142 is one of the most significant genetic variants associated with HUA in Asian populations [10,18]. Previous studies have identified that the minor T allele of *ABCG2* rs2231142 reached 27% in controls and 39.3% in HUA cases, much higher than that reported for Whites (11–12%) and Blacks (3%) [9]. In this Taiwan-based study, our current data confirmed that both males and females carrying the rs2231142 T allele had an associated increased HUA risk, which was consistent with other Asian population studies [8,16,36,54]. Consistent with our result, Lv et al. [55] demonstrated that the T allele of *ABCG2* rs2231142 was associated with increased HUA risk in diverse races, such as Asian, African, Caucasian, and New Zealand Pacific Islanders.

Our current study does have some limitations. First, we did not include all lifestyle risk factors that could impact HUA risk. Thus, more variables from lifestyle factors need to be included in future analyses to better investigate the association between *ABCG2* polymorphisms and other risk factors and the risk of developing HUA. Second, there is a possibility of a response bias considering that information was collected using questionnaires. Finally, the results of this study need to be validated in prospective studies with appropriate follow-up to validate our findings. Participants will need to provide information about their alcohol consumption, such as whether they regularly consumed alcohol and ethanol weight content.

## 5. Conclusions

In conclusion, our findings showed that alcohol consumption contributes, along with the *ABCG2* rs2231142 risk T allele, to increase HUA risk in Taiwan, especially in the female population. The findings suggest that a reduction in alcohol consumption is critical for high-risk patients with the *ABCG2* rs2231142 risk T allele. These results are important for understanding the joint effects of lifestyle risk factors and *ABCG2* rs2231142, and will contribute to the prediction of an individual’s risk for hyperuricemia depending on their gender.

## Figures and Tables

**Table 1:** Basic characteristics of the participants.

	Male (n = 41,265)			Female (n = 73,272)		
	Without HUA (n = 27,773)	HUA (n = 13,492)	*p*-Value	Without HUA (n = 70,018)	HUA (n = 3254)	*p*-Value
Age (years) (%) ^a^						
30–45	6023 (21.7)	3647 (27.0)	<0.001	15,315 (21.9)	398 (12.2)	
45–55	6945 (25.0)	3577 (26.5)		18,477 (26.4)	495 (15.2)	
55–70	14,805 (53.3)	6268 (46.5)		36,226 (49.4)	2361 (72.6)	<0.001
Current/ever alcohol consumption (%)						
No	24,598 (88.6)	11,267 (83.6)		68,709 (98.2)	3143 (96.6)	
Yes	3160 (11.4)	2215 (16.4)	<0.001	1277 (1.8)	109 (3.4)	<0.001
Current/ever smoker (%)						
No	12,041 (43.4)	5474 (40.6)		62,834 (89.8)	2898 (89.1)	
Yes	15,728 (56.6)	8017 (59.4)	<0.001	7168 (10.2)	356 (10.9)	0.203
Current/ever chewing betel nut (%)						
No	23,450 (84.5)	10,998 (81.6)		69,705 (99.7)	3232 (99.5)	
Yes	4286 (15.5)	2475 (18.4)	<0.001	210 (0.3)	17 (0.5)	0.034
Physical activity (%)						
No	15,815 (57.0)	7936 (33.4)		42,457 (60.7)	1858 (57.1)	
Yes	11,947 (43.0)	5551 (41.2)	<0.001	27,523 (39.3)	1396 (42.9)	<0.001
Body mass index (kg/m^2^) ^b^	24.77 ± 3.36	26.63 ± 3.62	<0.001	23.42 ± 3.64	27.05 ± 4.57	<0.001
Waist circumference (cm)	86.52 ± 9.06	91.04 ± 9.34	0.019	80.29 ± 9.53	88.98 ± 10.92	<0.001
Body fat percentage (%)	22.02 ± 5.31	24.76 ± 5.10	<0.001	31.65 ± 6.26	37.61 ± 6.52	0.084
Blood pressure (mmHg)	125.30 ± 17.12	128.29 ± 17.58	0.066	116.55 ± 18.34	127.41 ± 19.47	<0.001
Total cholesterol (mg/dL)	189.43 ± 34.34	196.83 ± 36.10	0.001	197.13 ± 35.71	209.89 ± 40.97	<0.001
Triglyceride (mg/dL)	124.51 ± 103.20	166.57 ± 141.91	<0.001	100.41 ± 70.69	161.95 ± 124.95	<0.001
HDL cholesterol (mg/dL)	49.13 ± 11.27	45.44 ± 10.33	<0.001	58.55 ± 13.19	50.39 ± 11.69	<0.001
LDL cholesterol (mg/dL)	120.02 ± 30.93	125.19 ± 32.31	<0.001	119.94 ± 31.59	132.01 ± 35.81	<0.001
Fasting glucose (mg/dL)	99.88 ± 25.67	98.26 ± 17.90	<0.001	93.62 ± 18.39	100.65 ± 21.43	<0.001

^a^ Comparisons of categorical variables were analyzed using the Chi-square test. ^b^ Continuous variables are summarized as mean ± standard deviation (SD) and were analyzed using Student’s t-test for normal data distributions.

**Table 2:** Genotypes and allele frequencies of *ABCG2* rs2231142 and risk of hyperuricemia in the study population.

Gene/SNP		Male (n = 41,265)					Female (n = 73,272)				
		Without HUA	HUA	*p*-Value ^a^	Risk of HUA		Without HUA	HUA	*p*-Value ^a^	Risk of HUA	
		(n = 27,773) (%)	(n = 13,492) (%)		OR (95% CI) ^b^	*p*-Value ^c^	(n = 70,018) (%)	(n = 3254) (%)		OR (95% CI) ^b^	*p*-Value ^c^
ABCG2 rs2231142				<0.001					<0.001		
	GG	14,366 (51.7)	5022 (37.2)		1		33,288 (47.5)	1102 (33.9)		1	
	TG	11,202 (40.3)	6545 (48.5)		1.67 (1.59–1.74)	<0.001	29,948 (42.8)	1626 (50.0)		1.64 (1.51–1.77)	<0.001
	TT	2205 (7.9)	1925 (14.3)		2.49 (2.33–2.67)	<0.001	6782 (9.7)	526 (16.2)		2.34 (2.10–2.61)	<0.001
Allele (%)				<0.001					<0.001		
	G	39,934 (71.9)	16,589 (61.5)				96,524 (68.9)	3830 (58.9)			
	T	15,612 (28.1)	10,395 (38.5)		2.49 (2.33–2.67)	<0.001	43,512 (31.1)	2678 (41.1)		2.34 (2.11–2.61)	<0.001

^a^ Analyzed by using the Chi-square test for association between genotypes of *ABCG2* rs2231142 and HUA. ^b^ OR = odds ratio; CI = confidence interval. ^c^ Logistic regression adjusted by age, BMI, hypertension, creatinine, TC, TG, HDL, and LDL.

**Table 3:** Interaction between alcohol consumption and *ABCG2* rs2231142.

Variables	Male (n = 41,265)		Female (n = 73,272)	
	OR (95% CI) ^b^	*p*-Value ^a^	OR (95% CI)	*p*-Value ^a^
Intercept	0.006 (0.003–0.015)	<0.001	0.016 (0.010–0.038)	<0.001
Age 45–55	0.851 (0.803–0.901)	<0.001	1.031 (0.902–1.178)	<0.001
Age 55–70	0.699 (0.665–0.736)	<0.001	2.508 (2.251–2.794)	<0.001
Current alcohol consumption	1.530 (1.443–1.623)	0.007	1.866 (1.530–2.276)	<0.001
BMI	1.162 (1.155–1.117)	<0.001	1.207 (1.198–1.213)	<0.001
Body fat percentage	1.105 (1.101–1.110)	<0.001	1.139 (1.133–1.145)	<0.001
Blood pressure	1.010 (1.009–1.011)	<0.001	1.027 (1.025–1.028)	<0.001
Total cholesterol	1.004 (1.002–1.006)	0.001	1.006 (1.002–1.009)	0.001
Triglyceride	1.002 (1.002–1.003)	<0.001	1.002 (1.002–1.003)	<0.001
HDL cholesterol	0.973 (0.970–0.976)	<0.001	0.951 (0.946–0.956)	<0.001
LDL cholesterol	1.002 (1.000–1.005)	0.067	1.005 (1.001–1.009)	0.007
Fasting glucose	0.993 (0.992–0.994)	<0.001	1.004 (1.003–1.005)	<0.001
rs2231142	1.707 (1.634–2.828)	<0.001	2.745 (2.219–2.880)	<0.001
Alcohol consumption ×rs2231142	2.183 (2.059–2.315)	<0.001	3.096 (2.403–3.988)	0.001

^a^ The odds ratios and *p*-values are assessed by combining with the interaction of current alcohol consumption. ^b^ OR = odds ratio; 95% CI = 95% confidence interval.

## Data Availability

The clinical data presented in this study are available on request from the corresponding authors. The genetic data from the Taiwan Precision Medicine Initiative are not publicly available. The authors confirm that, for approved reasons, some access restrictions may apply to the data underlying the findings. The data used in this study cannot be made available in the manuscript, the supplemental files, or in a public repository due to the Personal Information Protection Act executed by Taiwan’s government, starting in 2012. Requests for data can be sent as a formal proposal to obtain approval from the ethics review committee of the appropriate governmental department in Taiwan.
